# Our 3rd Early Career Researchers Peer Review programme is open for applications

**DOI:** 10.1038/s41467-022-31260-0

**Published:** 2022-06-23

## Abstract

Our peer review programme was launched in 2020 to support Early Career Researchers in building confidence to participate in peer review. The initiative has proved very successful and popular with both ECRs and editors and we are pleased to invite applicants to apply to our 2022 programme.

Too frequently, Early Career Researchers (ECRs) are not provided appropriate training for peer review nor do they receive acknowledgement for their contributions to peer review—often, they are not involved in the process at all. As peer review is one of the pillars of scientific communication and contributes to ensuring that high-quality, rigorous studies are published, we launched an initiative to introduce peer review to ECRs in 2020. We are now launching the third iteration of this programme to continue training and supporting this community, who represents the future of scientific research.

“86% of the ECRs reported that the experience increased their confidence to review and 91% said that it was helpful for their career development”.

Recently-appointed principal investigators sometimes reach their position with limited direct exposure to the peer review process. Such exposure is necessary not only to adequately evaluate the work of colleagues, but also to understand how one’s own research will be assessed. If PhD students and post-doctoral researchers rarely engage in direct interaction with editors, they may not gain experience of what a constructive peer review exchange with authors should be. Reviewers need to appreciate the authors’ arguments and consider that a manuscript should be seen as a defined entity in which the data support the main conclusions but do not need to address all the open questions in the field.

With this initiative, we aim to not only support ECRs through the peer review process, but also to provide insights into each of its steps. The programme begins with a webinar focused on the editorial peer review process and the guiding principles of assessing a manuscript and writing a quality peer review report. Participating ECRs are then paired with an editor based on their academic background and research expertise to assess and write a report on a manuscript that had been submitted to the journal. The editors guide the participants during the programme and provide feedback on their reports. As the process happens in parallel to the normal peer review of the manuscript, ECRs can also benefit from reading the reports of the other reviewers.

The 2020 pilot programme was carried out in partnership with Voice of Young Science—a branch of the UK based charity Sense about Science—and was a great success. It raised considerable interest within the broader ECRs community. Therefore, in 2021, we decided to widen this initiative and offer ECRs from all over the world the opportunity to participate through an open call. We also recruited editors from additional journals within Nature Portfolio as well as from BMC journals. Over 1500 ECRs applied to the programme and we received applications from diverse regions (40% North America, 41% Europe, 21% Africa, 4% South America, 10% Asia). We prioritized applicants at earlier stages of their research careers (mostly senior PhD students and post-docs) who previously had little or no experience in peer review, a selection process that allowed us to maintain the regional distribution of the participants. Around 450 ECRs were invited to the webinar and 110 ECRs who attended the webinar were selected to participate in the second phase of the programme and paired with 95 editors, who mentored their ECR through the assessment of a manuscript submitted to their journal. The questions asked during the two past webinars showcase the enthusiasm of the participants, and we compiled our answers in a guide for reviewers in the hope that they may be helpful to others. At the end of the programme, we collected feedback from both ECRs and editors. In 2021, 86% of the ECRs reported that the experience increased their confidence to review and 91% said that it was helpful for their career development; the editors reported that the comments received helped them reach an informed decision for 83% of the papers sent to the ECRs and in 91% of the cases they would consider inviting the ECR to review further manuscripts. Beyond supporting ECRs in reviewing the manuscript, the programme also offers recognition for the work done through our reviewer acknowledgement statement, should the ECR wish to be named.

Recruitment for the 2022 ECRs programme is now open until 10th of July. This year, we are aiming to increase the number of participating ECRs from geographical areas that are historically under-represented within the scientific community and from which we received a limited number of applications last year, such as Africa, South America and Asia. The candidates will be supported by our editors at the Open Access journals, *Nature Communications*, the Communications journals and the BMC journals. Spread the word, our editors are looking forward to meeting you!

